# Family carers’ narratives of the financial consequences of young onset dementia

**DOI:** 10.1177/14713012211009341

**Published:** 2021-04-20

**Authors:** Melanie Bayly, Megan E. O’Connell, August Kortzman, Shelley Peacock, Debra G. Morgan, Andrew Kirk

**Affiliations:** Canadian Centre for Health and Safety in Agriculture, College of Medicine, 7235 University of Saskatchewan, Saskatoon, Canada; Department of Psychology, 7235 University of Saskatchewan, Saskatoon, Canada; College of Nursing, 7235 University of Saskatchewan, Saskatoon, Canada; Canadian Centre for Health and Safety in Agriculture, College of Medicine, 7235 University of Saskatchewan, Saskatoon, Canada; College of Medicine, 7235 University of Saskatchewan, Saskatoon, Canada

**Keywords:** young onset dementia, financial consequences, family caregivers, dementia, qualitative

## Abstract

Individuals with young onset dementia and their families face unique challenges, such as disruptions to their life cycle and relationships and a dearth of appropriate supports. Financial consequences have also been noted in the literature yet have not been explored in-depth. The purpose of this research was to qualitatively explore carers’ experiences of financial consequences resulting from the young onset dementia of a family member and how these consequences may be managed. Eight carers (7 women and 1 man) provided a written online narrative about their journey with young onset dementia and any financial consequences experienced, with open-ended prompts to elicit details not yet shared. Narratives were inductively coded and analyzed using a thematic narrative approach. Carers described a voluntary or involuntary end to employment for the person with young onset dementia around the time of diagnosis. This engendered ongoing and anticipated financial consequences, combined with the need for carers to balance employment with the provision of care (which often meant early retirement for spousal carers). Common themes were tension between the needs to provide care and earn income, altered financial prospects, costs of care, and lack of available and accessible supports to ameliorate financial consequences. Findings illustrate the reality of financial consequences across the trajectory of young onset dementia. These consequences may manifest differently for spousal and child carers and are not being adequately addressed by existing supports.

## Introduction

Young onset dementia refers to the onset of dementia symptoms prior to the age of 65. The prevalence rate of young onset dementia ranges from 67 to 81 cases per 100,000 individuals aged 45–65 years, with fewer cases occurring among younger people (Vieira et al., 2013; Werner et al., 2009). Reviews on prevalence by sex are lacking, although a recent meta-analysis suggests that females may be at higher risk for one variant of frontotemporal dementia (Curtis et al., 2017). No sex difference in prevalence is evidenced in the patients diagnosed in our rural and remote memory clinic, where M.E.O. and A.K. practice clinically (Wong et al., 2020). Although Alzheimer’s disease remains the most common dementia diagnosis among both older and younger adults, individuals with young onset dementia are more likely than persons with later onset dementia to be diagnosed with atypical forms such as vascular and frontotemporal dementias (Harris, 2004; Vieira et al., 2013). Partially because of this different etiological profile, predominant symptoms of young and later onset dementia differ. Individuals with young onset dementia often have higher levels of hyperactivity or apathy and are more likely to initially present with behavior and personality changes or language disturbances rather than memory loss (Bakker et al., 2014; Harris, 2004).

Along with clinical and etiological differences, the experience of young onset dementia appears to be unique. Accurate and timely diagnosis can be a significant challenge because dementia is not often associated with younger people, young onset dementia has a more varied differential diagnosis, and presenting symptoms are often neuropsychiatric (Harris, 2004; Flynn & Mulcahy, 2013; Mendez, 2006). Delayed diagnosis can be very stressful for people with young onset dementia and their families and hinders their ability to obtain needed supports (Allen et al., 2009; Roach et al., 2008; van Vliet et al., 2010, 2011). Indeed, early and correct diagnosis has been highlighted by patients and carers as the most important need related to young onset dementia (Armari et al., 2012).

Adequate support may not be provided upon a diagnosis of young onset dementia (Flynn & Mulcahy, 2013; Johannessen & Möller, 2011), and lack of appropriate supports and services persists post-diagnosis. The authors of a recent systematic review on age-appropriate services for people with young onset dementia concluded that such services remain “fragmented, geographically dispersed, variable, and often short-term due to project-based commissioning” (Mayrhofer et al., 2018, p. 939). Services and supports tend to be geared toward older adults and are perceived by people with young onset dementia and carers as inappropriate for younger persons (Green & Kleissen, 2013). Perceived inappropriateness of services may lead to their underuse, as well as a sense of isolation for persons with young onset dementia (Clemerson et al., 2014) and perceived discrimination by carers (Werner et al., 2020). Carers of persons with young onset dementia report numerous unmet skill-based, educational, and support needs (Bakker et al., 2014; Ducharme et al., 2014; Green & Kleissen, 2013) and dissatisfaction with the amount and quality of formal supports (Millenaar et al., 2018).

While changes in life trajectory and familial relationships are characteristics of later onset dementia as well, the roles and responsibilities of younger persons typically differ compared to older adults: employment demands, children at home, and ill or older parents (Ducharme et al., 2014). Young onset dementia may also engender feelings of profound disruption to the life cycle as individuals consider themselves (and are considered by others) too young to have a dementia diagnosis, feel prematurely aged, and must grapple with the loss of an expected future (Clemerson et al., 2014). Multiple aspects of identity may be affected, including employment role, which can elicit grief, perceived loss of self, and decreased self-esteem (Harris, 2004; Rabanal et al., 2018; Roach et al., 2008). Changing familial roles and relationships also occur, including changes to the sexual and spousal relationship, increased household, parenting, and caregiving responsibilities for spouses and increased responsibilities, caregiving, and the perceived loss of a parent on the part of children (Allen et al., 2009; Aslett et al., 2019; Busted et al., 2020; Ducharme et al., 2013; Flynn & Mulcahy, 2013; Gelman & Rhames, 2020; Harris, 2004; Holdsworth & McCabe, 2018; Wawrziczny et al., 2016). These changes may be distressing to families and lead to increased conflict around responsibilities (Kimura et al., 2015; Roach et al., 2008, 2016).

Finally, financial consequences uniquely impact families affected by young onset dementia. Early symptoms may negatively impact work performance and relationships, eventually leading to reduced work hours, resignation, or job loss that cause financial difficulties and feelings of being a financial burden (Busted et al., 2020; Evans, 2019; Harris, 2004; Johannessen & Möller, 2011; Kimura et al., 2015; Roach et al., 2016; Rose et al., 2010). At the point of job loss or resignation, symptoms may prevent persons with young onset dementia from finding or maintaining another job (van Vliet et al., 2011). Loss of employment benefits may further increase negative consequences for families (Green & Kleissen, 2013). Leaving work unexpectedly due to performance issues can also impact one’s ability to obtain post-employment government financial assistance (Roach et al., 2016). The financial impacts of this may be significant as some individuals with young onset dementia are at the height of their career and have financial obligations such as mortgages or children’s educational expenses (Harris, 2004; Roach et al., 2008). Moreover, spouses often reduce employment to fulfill a caregiving role (Flynn & Mulcahy, 2013; Kimura et al., 2015; Roach et al., 2008; Werner et al., 2020), or begin working for the first time in low-paid jobs to provide needed income (Allen et al., 2009). Need for the carer to maintain employment is a source of significant stress, and spouses who remain working and have children at home may experience the greatest difficulties (Ducharme et al., 2013; Roach et al., 2016). Despite the financial difficulties described above, there may be little government-provided financial support for care (e.g., pensions and paid respite care) due to the young age of dementia onset (Flynn & Mulcahy, 2013). Werner et al. (2020) characterized the exclusion of younger persons with dementia from such supports as a form of structural stigma that increases families’ financial difficulties.

Although these negative financial consequences from young onset dementia have been noted in the literature, they have not to date been an empirical focus. Moreover, little is known about how families cope with negative financial consequences and what they have in terms of support; Mayrhofer et al. (2018) noted that post-diagnostic support regarding employment, legal, and financial issues is crucial but currently under researched. The purpose of this research was therefore to qualitatively explore how family carers of persons with young onset dementia understand their experiences, with a focus on financial consequences and how they may be ameliorated by supports.

## Methods

The current research was a qualitative narrative study (Riessman, 2008), with the goal of exploring the written stories of family carers to persons with young onset dementia with a focus on financial consequences. The feasibility of using online generation of narrative data with this population was also explored, to inform future work. This research was reviewed and approved on ethical grounds by the University of Saskatchewan REB (#17-423).

### Participants

The only inclusion criteria was being a family carer to a person with young onset dementia. Participants were recruited via online advertisements through Facebook and the Alzheimer Society of Saskatchewan that included a link to the online survey. Eight participants completed this survey and shared their experiences with young onset dementia in narrative form. These participants were primarily urban Canadian women (one respondent identified as male), with a mean age of 52. Most (five) were spouse/partners to the person who had been diagnosed with young onset dementia, whose mean age was 55. The majority of participants reported their family had some form of health insurance, and all participants except Nancy, Janice, and Audrey indicated that they had the financial resources to meet their daily needs. Detailed participant information can be viewed in Table 1.

**Table 1.** Participant demographic information.

Carer^a^	Age	Sex	Kinship	Country	CR^b^ age	CR age at symptom onset/diagnosis	Diagnosis (all young onset)	Health insurance
*Nancy*	37	F	Niece	Rural Canada	52	46/49	AD	None
*Janice*	55	F	Partner	Urban U.S.	55	40/48	LBD and PD	Veteran’s benefits for CR and none for family
*Karen*	49	F	Child	Urban Canada	77	62/late 60s	AD	Sunlife
*Ella*	33	F	Child	Urban Canada	57	54/55	AD	CR covered by LTD
*Audrey*	57	F	Partner	Rural Canada	63	60/61	AD with aphasia	Provincial and Blue Cross
*James*	57	M	Partner	Urban Canada	57	52/54	Dementia	None
*Maryam*	62	F	Partner	Urban Canada	65	58/61	Posterior cortical atrophy	Blue Cross x 2
*Emma*	62	F	Partner	Urban Canada	63	57/59	AD	Blue Cross

^a^Participant names are pseudonyms.

^b^CR = care recipient.

### Data generation

After providing informed consent, participants were asked to complete the demographic questions represented in Table 1. They were then asked the following question to elicit a narrative description of their experiences:

*We would like to understand what the journey of young onset dementia has been like for you, your loved one, and the rest of your family. We are especially interested in understanding any employment and financial consequences that have resulted from symptoms and the diagnosis, and how your family has coped with those consequences. Starting wherever you would like to start, please share your young onset dementia story in as much detail as you are comfortable with:*

Participants typed their story into an expanding, unlimited text box; they could also respond to specific open-ended questions about the financial consequences of their experiences. We asked participants to respond to these questions if they had not already shared these details in their narrative or wanted to elaborate further. Questions were developed based on prior research and the clinical experience of MEO, and covered changes to their own and the care recipient’s employment, support service access and needs related to financial aspects of young onset dementia, and changes to their well-being and quality of life as a result of financial consequences of young onset dementia.

### Analysis

Data were analyzed using a thematic narrative approach, where the focus of analysis is primarily on the content of the narratives rather than their form or function (Riessman, 2008). Narratives were initially read several times, and the overall tone and flow of each was noted. Three chronological phases were clear in participants’ narratives: pre-diagnosis, around the time of diagnosis, and managing life post-diagnosis. Inductive coding was performed with data pertaining to each of these chronological phases, to illustrate what was salient during each period. Codes represented the most meaningful features being communicated within each data element and were organized into themes representing common patterns across narratives (Braun & Clarke, 2006). Coding and analysis was focused around the financial consequences of young onset dementia. Although the main unit of analysis was participants’ story narrative about their experiences with young onset dementia, responses to our specific questions were analyzed when they provided elaborative detail.

### Findings

Carers’ narratives differed in emotional tone and presentation of how their family has been affected by young onset dementia, beyond the financial consequences (see Table 2 for a brief snapshot of each narrative). Themes illustrating financial consequences represent elements common across multiple carers’ stories: involuntary job loss, early retirement, tension between employment and caregiving role, altered financial prospects, anticipated and current costs of care, and minimal formal supports. These are delineated below.

**Table 2.** Overall tone and focus of carers’ narratives.

Carer*	Description of narrative	Illustrative quote
*Nancy (niece)*	The main tone of Nancy’s narrative was frustration. Her story focused primarily on the consequences of her aunt’s dementia on their family and their difficulties as carers due to the lack of appropriate formalized supports.	*“I feel frustrated most days… fielding calls from [Aunt] and from the nursing home… dealing with drama to medical…I’m finding it hard to get things done with the added responsibility of [her] well being. I stress often and worry am I doing the right things?”*
*Karen (child)*	Karen’s narrative unfolded primarily through answers to our specific questions and focused entirely on the significant financial, social, and emotional impacts she perceived her mother’s young onset dementia had on her and her sibling. Karen’s narrative conveyed a sense of despair at their situation.	“*The situation has been personally and financially devastating to myself and my sibling… I am emotionally damaged and honestly don’t know if I will ever be the same.”*
*Ella (child)*	Ella’s narrative was a very matter-of-fact presentation of her mother’s symptoms, diagnosis, and ongoing management of young onset dementia. She presented her and her spouse as active carers who do what is required to meet her mother’s needs.	*“We moved her back to [City] immediately, renting a condo for her on a one year lease. After the year, it was apparent that she could not live on her own without supervision, as she would forget to eat, was lonely, etc., she we chose to move her in with us.”*
*Janice (partner)*	Janice depicted her experiences with young onset dementia as a battle. She believed her husband’s dementia was caused by his military service/exposure to chemicals, and her narrative reflected anger at the lack of formal but also informal support she was receiving despite advocating for her family.	*“Again, our family and close friends have turned their backs on us, therefore I have reached out to all known sources for aid and assistance; however, none has been provided. Not even so much of a courtesy response from our local government offices.”*
*Audrey (partner)*	Audrey depicted her experiences with her husband’s young onset dementia as challenging, but her narrative did not convey a sad, frustrated, angry, or despairing tone. She described in detail how her husband has changed since his symptoms began and how their lives continue to change as his health declines.	*“Because of my husband’s career choices, he never had ‘time’ for hobbies. Work has been his only interest from the time he was a child, but we no longer own a farm and he could not be able to do the work even if we still had it. My husband is physically strong for short bursts, but the consistent strength required to work with animals is past him now.”*
*James (partner)*	James’ narrative was one of cautious optimism in the face of challenge. Although he depicted their situation with young onset dementia as difficult, it was clear he took his role as a carer very seriously and felt that his family was managing well despite his wife’s diagnosis and the financial consequences that stemmed from it.	*“That resulted in her getting her license back renewing some of her self-esteem and increasing her belief that her condition is mild and that she can beat it. She calls it denial and I call it hope. I continue to give her my full support and keep her life as normal as possible and make her feel that she is still in control.”*
*Maryam (partner)*	Maryam described in detail how much their lives have changed since the onset of her husband’s dementia symptoms. A significant focus in her narrative was the degree to which her world had shrunk and the loneliness she felt at directing all of her energy toward caring for her husband.	*“I am much more sedate now, try to get [Husband] out walking if he can and will. I cannot just leave him alone to go out to leisure or sport activities, therefore I do less…. Family and friends visit less, they are unsure how to deal with [Husband’s] illness. This journey [Husband] and I are in is very lonely.”*
*Emma (partner)*	Emma explained the onset of her husband’s symptoms and period of diagnosis in detail. The dominant tone of Emma’s narrative was one of fatigue as she described her worries about finances and the things she has lost or given up as her husband’s young onset dementia progresses.	*“I miss adult conversations with him more than anything I think. I tell him less and less about things because I know he will forget it anyway. He is still a very kind and gentle man. For that I am grateful but a lot of the time I’m just resentful, not at him but at life in general.”*

### Pre-diagnosis symptoms and voluntary/involuntary job loss

All but one carer (who relied more on the questions to tell their story) began their narrative by describing the onset or cause of dementia symptoms, which included slurred speech, difficulty word-finding, memory difficulties, confusion and disorientation, difficulty performing daily tasks, difficulty making and following plans, loss of focus, fatigue, tremors, personality changes, and lack of interest in finances. Several carers narratively linked these symptoms and eventual diagnosis to a specific cause: a stressful period of life, multiple concussions, or exposure to hazardous chemicals and heavy metals. Janice’s narrative was structured around her conviction that her spouse had incurred dementia as a result of occupational exposures:

*My husband Young Onset Lewy Body Dementia is a result of serving our country and being exposed to hazardous chemicals without the necessary personal protective equipment. Of course, today the hazardous chemicals are now banned, and the personal protective equipment is a mandate by the Occupational Health and Safety Administration. However, I have been denied Veterans Disability Compensation.*

For at least three families (timing was not clear in one narrative), financial consequences began during the pre-diagnosis period as a result of symptoms. Maryam described a long period during which symptoms were present, but her partner continued to work:

*[Partner] became overly tired, easily overwhelmed, memory poor, no mental power… I encouraged the retirement as he seemed tired, and he experienced a loss of mental power very noticeable to me. Post retirement, he chose to do a summer job... he struggled with the math, and basically the job became too much. I began to worry something was up. His hand began to tremble and his ability to focus long periods on visuals was weakening.*

Maryam’s partner continued to work part-time for several years but was entirely unable to work by age 59, still prior to his formal diagnosis. Maryam also retired early because of her partner’s health, shortly after his final exit from the workforce.

Two other carers described how the person with dementia’s symptoms led to job loss. Emma explained that her partner lost his job due to his memory difficulties, although he started another and worked until after his diagnosis. Nancy recounted how her aunt’s symptoms were misattributed to drug or alcohol use, which led to her being fired:

*After he [son] came home she went back to work… her speech starts to go and it was either slurred or it was mixed up wording. Her co workers and employer blamed drug use… she was not a drug user. Her own family dr accused her of using drugs and alcohol. Then she lost her job, she tried getting medical help prior to this and was told by her family doctor to just stop drinking…*

Similar struggles to receive an accurate diagnosis were reflected in other carers’ accounts.

### The journey to diagnosis and end of employment

Carers told of family members initiating memory assessment, due to concerns about observed symptoms. The journey to diagnosis was presented as difficult and nonlinear, with some families recounting delay from healthcare professionals who attributed symptoms to alternate causes (e.g., drugs/alcohol and mental health) and others describing a drawn-out period of tests and travel. Ella wrote how family in her mother’s new city of residence reached out with concerns about her memory and confusion, which prompted Ella’s family to seek a diagnosis:

*We brought her back to [city] to speak with our family doctor, and seek help. We initially had her looked at by a doctor at the Neurology Clinic, but was recommended that she suffered only from depression and boredom, due to being off work and recovering from my dad’s death. We seeked out further evaluation and went through more intense evaluation, occurring on multiple occasions at the Memory Clinic in [other city]. After several visits, she was diagnosed in June.*

Three carers described seeking second opinions upon hearing the diagnosis. Audrey’s partner’s diagnosis from his family GP came as a shock, despite symptoms:

*My husband stormed out of the office, and began to ask for a second opinion. I found a neurologist who examined my husband, and told him he felt the diagnosis had been a mistake. We attended a clinic in Alberta to get a CAT scan quickly.* ^1^ *This seemed to confirm the diagnosis, and dashed the hopes the neurologist had given us… we spent the next several months going through different assessments to regain that hope the initial neurologist had given us to no avail. This included spending all of our savings on a trip to the Mayo Clinic, where my father-in-law felt the best tests were available in the world. All of this confirmed the diagnosis of Young Onset Alzheimer’s with Aphasia.*

While the process of diagnosis was therefore costly for Audrey and her partner, the diagnosis also meant the loss of his license and therefore his job: *“My husband lost his job the day he was diagnosed- immediately. He lost his drivers license, which cost him his job and his freedom.”*

Indeed, for the people with young onset dementia who were still working, it was the period around diagnosis that marked the shift to retirement in carers’ narratives. Emma wrote that her partner stopped working shortly after diagnosis: *“…he was making silly mistakes at work and people were noticing so we made the decision for him to stay home.”* Instead of being symptom- or performance-related, James framed his partner’s cessation of part-time employment as a way to slow her cognitive decline and preserve health:

*As a result of her diagnosis we immediately took steps to remove the stress in her life, give her more hours of sleep, keep her physically active and reviewed her health and nutrition… Following her diagnosis, in spite of the fact that she felt she could still be employed and successful, we decided to end her part-time job and eliminated all thoughts of future employment. This was all done to reduce her stress and simplify her life. Both of these have been very beneficial to her…*

The ability for his spouse to stop working and his own ability to support her despite financial consequences were central in James’ narrative.

### Managing life post-diagnosis and the ongoing financial consequences

While carers’ descriptions of pre-diagnosis and the period around diagnosis centered around *job loss* and *early retirement* for the person with young onset dementia, the rest of their narratives depicted ongoing impacts (financial and otherwise) that resulted from the end of employment and their caring role. A predominant theme was *tension between carer employment and caregiving*. Adult children primarily tried to juggle caregiving with employment; Ella stayed home to provide care after her maternity leave, eventually hiring in-home help to support her part-time return to work. This was not an option for Nancy, who stated she could not take time off work with two children to support. Karen described how difficult balancing care and work had been for her and her sibling:

*My job status changed but I have received multiple warnings at work due to missing so much to help my parent with urgent situations, medical appointments, and managing her affairs. My sibling quit their job due to all of these things…*

Karen did not foresee any easy solution for their family to resolve the tension between caring and employment.

Most spouses who were still working also retired early, in order to manage caring demands. Emma explained:

*I quit work in December 2017 because I wasn’t comfortable with him being alone all day. He plateaued for awhile after that as we got used to spending so much time together but gradually he understands less and less of day to day activities…I would have liked to work longer but my husband would not hear of having a “babysitter” in my place. It would be nice to have a choice in whether I have to quit work or not.*

As in Emma’s passage above, ambivalence and tension between work and caregiving was often present even when spouses retired early. This tension was presented clearly by James, who was laid off and chose not to return to work in order to care for his wife:

*Financially, this situation is a blow for us. Although we have always lived lean we currently don’t have a lot of margin to accommodate large emergencies. I have not applied for government assistance because I don’t know if I should or if I would even be successful. I have not looked for other employment but I may in the near future. I am always torn between the daily stress of caregiving and the stress of being in the workforce. The question “What should I do”, causes a lot of stress and concern at times because there is no real answer to that question… at 57 years of age, my desire to be a good husband, who is giving my terminally-ill wife the happiest and longest life possible is in conflict with my belief that I need to be working to continue to build a nest-egg and a career.*

The only spouse who continued some work was Audrey, who also described tension between her paid work and caring roles despite giving up her employment contract to work only on call:

*Now, I am on call and will work on days that my husband is doing well. My boss doesn’t truly understand that I need to cancel bookings, sometimes. I, too, have issues dealing with the fact I can’t work anymore than I am.*

The young onset of dementia therefore caused role tension for carers, regardless of how they negotiated the balance between work and caregiving.

Spousal carers generally presented their situation as one of *altered financial prospects*. An early end to employment meant changing lifestyle, giving up on future dreams, adjusting to lost income and diminished retirement savings, and trying to meet financial needs under new circumstances. Janice framed her partner’s diagnosis as having a “huge tsunami ripple effect” in their family’s lives as she tried to raise their three young children:

*My husband’s disability has caused a tremendous financial burden. Both of us were in the prime of our careers. Having the rug pulled out from us financially, mentally, emotionally, and physically is more than I ever could have imagined… After living in my home for 16 years, I have been faced with foreclosure twice in 2 years, and the next foreclosure notice will inevitably occur…*

Three of these carers reported they struggled to meet daily financial needs. Others whose situations were more stable still described living off of their savings, living frugally, and worrying about finances. Emma wrote about dipping into retirement savings for a recent expense and explained:

*Part of me knows that we will be fine financially but a larger part worries that there isn’t enough there to last another 30 years. I worry a lot about money. I’m always trying to find ways to save which isn’t easy with four beautiful grandchildren that we love to spoil. What usually happens is we spend on them and not on ourselves. I’m tired of trying to balance our statement but mostly I’m tired of not having anyone to share my fears and apprehensions about it with.*

Even carers who appeared to a have a financial cushion wrote about financial worry and reduced spending or restriction.

Both adult child and spousal carers described *anticipated and current costs of care* within their narratives, which had a financial impact. Child carers, like Karen, described spending on formal care and informal expenses for their parent:

*I had to lend a large amount of funds to my parent to cover expenses of assisted living before they could qualify for a nursing home, and this devastated me financially, emotionally, in my relationship with my spouse which was almost destroyed, and caused me chronic insomnia.*

Ella moved her mother back to their city and rented a condo for her before bringing her to live in their own home. Nancy described having to sell their mother’s home, which had been built by her grandfather, so she could pay her mother’s debts and costs of living. A few spouses also wrote about costs, mainly concern about future costs. Maryam described her efforts to balance needs with costs:

*I do not like leaving my husband alone, the guilt is huge. Also safety looms with me continually, I worry about his emotional state never mind mine. How do I attend support groups and leave my husband alone??? Makes no sense to me, hire a sitter??? Costs are outrageous for evening help… I continually watch our money. Spend very little as worried I will need it for care. We moved to a condo which I am grateful for but the tiny space has definitely impacted social gatherings.*

Maryam highlighted in her narrative how her social world had shrunk significantly, which she felt was partially related to the financial consequences of young onset dementia.

Overall, carers felt there were *minimal formal supports* for dealing with the financial consequences of young onset dementia. James, Janice, and Audrey described loss of extended/corporate healthcare plans with the end of employment. Janice, who lived in the United States, described a dearth of health insurance and financial supports that affected her well-being:

*There’s no doubt that by me not having health insurance benefits for years that I very well could have a chronic illness.*

Audrey wrote of ongoing challenges with their limited health coverage, despite being in Canada:

*I wish I could say we were better prepared for this, but I believed we would be covered by our health plan. Unfortunately, our corporate health plan was cancelled immediately upon my husband losing his job. Even though we renewed with the same health coverage, they specifically would not cover anything to do with Alzheimer’s. His prescription costs approximately $130/month with CPP-D being my husband’s only source of income maxing out at $1500/month. Finances are a big concern for me… Because my husband is utilizing CPP-D he will have a substantially reduced CPP after age 65. No banks or financial institutions have offered any assistance, and any business we were dealing with have wanted every penny immediately upon notification of my husband’s diagnosis. Because we were incorporated with my husband’s truck and farm, this diagnosis has cost us literally tens of thousands of dollars.*

As mentioned in Audrey’s passage above, CPP-D (Canadian Pension Plan- Disability) is a financial support theoretically available to persons with young onset dementia who are under the age of 65 and meet contribution requirements. Audrey reported that her partner was receiving these benefits with the assistance of his physician, and Emma’s spouse was considering applying for them. James, however, described how his wife was denied CPP-D:

*We applied for the CPP disability benefit and we were declined because her qualification date was in 2004 and we were required to prove that the condition existed back then (which is very, very difficult to do).*

James stated that they had not accessed any services or formal supports to help with financial challenges and that he did not know of any that could help them. Similar sentiments were echoed by Karen, who perceived a lack of education and supports to help with financial consequences of young onset dementia:

*I am not aware of any that we could have accessed, and I have spent a LOT of time looking for this kind of information… The services that help financially that I have heard of only apply when someone is dying or when being cared for full time in the carer’s home, and neither of these were our situation.*

Similarly, Nancy (caring for her aunt) wrote *“No there’s no supports.”*

These carers primarily illustrated situations in which they were falling through the cracks of government support systems. The subsidization of care costs (medication, home care, and respite) was one way other than CPP-D through which carers felt financial consequences were being ameliorated. Maryam wrote:

*[Partner] just recently is attending a day program (4 hours) which is subsidized by government, we would have difficulty affording memory care at $35.00 per hour, or respite at $180.00 per night! Absolutely no way we could afford a private memory unit at $5000.00 per month.*

Emma expressed similar appreciation for their subsidized day program, which she felt was affordable. James indicated he would be relying on their provincial healthcare system for services needed in the future such as home care, respite, and long-term care.

Some carers made specific suggestions for improvement to services and supports to help address the financial consequences of young onset dementia. These included subsidization of memory care units and increased availability of funded home care evening assistance, access to employment insurance to take care of a family member, appropriate and affordable healthcare coverage unaffected by the dementia diagnosis, and further reductions to the cost of day programing. For example, Emma wrote: *“Keeping the cost down for day programs so that he could attend more often, would have possibly allowed me to work longer.”* Two carers also suggested that more education on available services/supports was important; James wrote:

*If there is any way to reassure families facing the challenges of this disease that there are safety nets in the future and what the safety nets are. It’s complex and all-consuming situation at times and only gets worse as the disease progresses.*

## Discussion

Family carers highlighted a number of financial consequences from young onset dementia. Their narratives followed a similar progression, with a voluntary (retirement) or involuntary (fired due to symptoms) end to employment for the person with dementia occurring pre-diagnosis or around the time of diagnosis. This was followed by descriptions of ongoing and anticipated financial challenges from the end of employment, combined with the carer balancing employment and caring roles (which tended to mean early retirement for spouses). Common across narratives were depictions of tension between the need to provide care but also bring in income, altered financial prospects, current and anticipated costs of care, and lack of accessible, available services or supports to help ameliorate financial consequences. It was also clear that financial consequences had further impacts on carer well-being, even for families who were currently able to meet their financial needs and had savings for the future. Thematic narrative analysis of family carers’ written narratives about young onset dementia highlighted these shared elements, despite the uniqueness of each carer’s experiences.

Findings offer an in-depth examination of the financial consequences of young onset dementia as understood by family carers, previously missing from the literature. These findings are congruent with past research that identified job loss and the need for early retirement of the person with dementia (e.g., Evans, 2019; Harris, 2004; Johannessen & Möller, 2011; Kimura et al., 2015; Roach et al., 2016; Rose et al., 2010), the need for spousal reduction/end of employment (Flynn & Mulcahy, 2013; Kimura et al., 2015; Roach et al., 2008), the financial impact this has on families (Harris, 2004; Roach et al., 2008), and stress from balancing the need for income with caring responsibilities (e.g., Ducharme et al., 2013; Roach et al., 2016). Our findings emphasize that carers to family members with young onset dementia face financial challenges, which can be significant and were described by several carers as “devastating.” They also illustrate ways in which a diagnosis of young onset dementia may result in altered financial prospects: families may change future plans, sell a family home, reduce spending, and face difficulties meeting daily needs.

Our findings also suggest the financial consequences of young onset dementia may differ between child and spousal carers. Costs of care (paying for their parent’s residence, moving them in, spending money on their activities, and paying for care) were more salient in children’s narratives compared to spouses. While adult child carers recounted short-term financial impacts, they did not describe altered prospects such as concerns around saving for future costs and care. Moreover, spouses in our research primarily retired after their partner’s diagnosis, whereas adult children had less ability to cease work and therefore navigated tensions between employment and caregiving differently. In past research on young onset dementia where financial consequences have been discussed, participants were primarily spouses of the person with dementia. Where researchers have interviewed both spousal and adult child carers, the relationship of the carer to the person with young onset dementia was not considered in analyses, and the research was not focused specifically on financial consequences (e.g., Flynn & Mulcahy, 2013; Kimura et al., 2015; Ducharme et al., 2014). Aslett et al. (2019) recently explored the impact of having a parent with young onset dementia with their five adult child participants. Although they listed subthemes such as giving up career, juggling responsibilities, and life on hold, financial consequences were not delineated in their analysis. Teasing apart the financial impacts on spousal compared to adult child carers of persons with young onset dementia may be a useful avenue for further research.

Finally, findings highlight the limited formal services and supports available. Adult child carers reported no available financial supports, and supports reported by spousal carers (e.g., CPP-D) had eligibility requirements that could be difficult to achieve in the context of young onset dementia. Canadian care recipients were not old enough to be eligible for their regular pension plan income; similar findings were reported by Flynn and Mulcahy (2013) within an Irish context. Moreover, the cessation of employment health benefits meant that some families lost coverage required for ongoing health needs. Canadian participants did however describe the subsidization of care costs (medication through health plans and respite through provincial health) as helpful for ameliorating financial consequences. Otherwise, they described little besides reduced spending that helped them cope with financial consequences of young onset dementia, suggesting that they were largely reliant on savings/continued income and were on their own.

### Implications

These findings highlight a major gap in financial supports for persons with young onset dementia. Lack of structural financial support (e.g., no pension and restriction to other entitlements like respite care) has been documented for persons with young onset dementia (Flynn & Mulcahy, 2013). Armari et al. (2012) found that 44% of patients and 33% of carers felt improvements to financial supports for families affected by young onset dementia are needed, and access to such supports has been identified by carers as one of their greatest unmet needs (Ducharme et al., 2014). Our findings are consistent with the clinical experiences of M.E.O. and A.K., who have diagnosed numerous persons with young onset dementia who report a recent history of terminated employment or premature retirement. A lucky few receive a diagnosis while on short-term disability leave and are able to receive long-term disability benefits post-diagnosis. We suggest advocating for policy change to open access to long-term disability benefits retroactively for all who receive a diagnosis of young onset dementia within 2–3 years post-termination or early retirement. Such a policy change would address some of the financial concerns of families of persons with young onset dementia. It would also match our understanding of the underlying diseases causing young onset dementia as progressive in nature, by acknowledging the role of undiagnosed cognitive impairment in persons’ termination or premature retirement.

### Limitations

As noted earlier, we had only one male participant in our small sample. While congruent with the larger literature in which the majority of carers to persons with dementia are female, our sample limited our ability to analyze how sex and gender may intersect with carers’ experiences of the financial consequences of young onset dementia. For example, whether male and female spousal carers are equally likely to deal with caring demands by retiring early, or how gendered roles and structural factors may affect the consequences of premature employment cessation, are unknown. Questions of how sex and gender may influence the financial consequences of young onset dementia remain to be explored empirically and would address calls to examine sex and gender in the context of dementia and neurodegeneration research (e.g., Bartlett, et al., 2018; Tierney et al., 2017).

Additionally, our method of data generation had both strengths and limitations. Eliciting online written narratives did not allow for the same degree of co-construction of narratives that would have occurred with interviews. This was a limitation in that when participants briefly mentioned something of interest in their written narrative, we were unable to engage in dialogue that would elicit further detail and reflection. Speaking is also less labor-intensive than typing, and this may have limited some of the written narratives we received. We were, however, pleased by the time and effort participants put into sharing their experiences as most narratives were quite rich in detail.

## Conclusions

Our findings suggest that from pre-diagnosis to ongoing life with young onset dementia, families experience financial consequences that begin with, and stem from, the loss of employment or early retirement of the person with dementia. These consequences may manifest differently depending on carers’ social location (e.g., age and generation, geographic location, and socioeconomic status) and context (e.g., degree of family assistance and retirement planning) and are not adequately addressed by formal supports and services. This research is the first to our knowledge that focuses specifically on the financial consequences of young onset dementia, as perceived by family carers. This is an aspect of the experience that has been understudied to date yet has significant impacts on the lives of persons with young onset dementia and their families.

## Note

1 Presumably this clinic was private, unlike the majority of health services in Canada which are funded publically.
